# Mismatch Negativity to Threatening Voices Associated with Positive Symptoms in Schizophrenia

**DOI:** 10.3389/fnhum.2016.00362

**Published:** 2016-07-15

**Authors:** Chenyi Chen, Chia-Chien Liu, Pei-Yuan Weng, Yawei Cheng

**Affiliations:** ^1^Institute of Neuroscience, National Yang-Ming University, TaipeiTaiwan; ^2^Department of Psychiatry, National Yang-Ming University Hospital, YilanTaiwan; ^3^Department of Rehabilitation, National Yang-Ming University Hospital, YilanTaiwan

**Keywords:** schizophrenia, emotional salience, voices, mismatch negativity, receiver operator characteristic

## Abstract

Although the general consensus holds that emotional perception is impaired in patients with schizophrenia, the extent to which neural processing of emotional voices is altered in schizophrenia remains to be determined. This study enrolled 30 patients with chronic schizophrenia and 30 controls and measured their mismatch negativity (MMN), a component of auditory event-related potentials (ERP). In a passive oddball paradigm, happily or angrily spoken deviant syllables *dada* were randomly presented within a train of emotionally neutral standard syllables. Results showed that MMN in response to angry syllables and angry-derived non-vocal sounds was significantly decreased in individuals with schizophrenia. P3a to angry syllables showed stronger amplitudes but longer latencies. Weaker MMN amplitudes were associated with more positive symptoms of schizophrenia. Receiver operator characteristic analysis revealed that angry MMN, angry-derived MMN, and angry P3a could help predict whether someone had received a clinical diagnosis of schizophrenia. The findings suggested general impairments of voice perception and acoustic discrimination in patients with chronic schizophrenia. The emotional salience processing of voices showed an atypical fashion at the preattentive level, being associated with positive symptoms in schizophrenia.

## Introduction

Schizophrenia, a chronic and disabling brain disorder, has three categories of symptoms: positive, negative, and cognitive symptoms. Hearing voices is the most common type of hallucination associated with positive symptoms. Deficits in the ability to recognize emotions from vocal expressions are treatment resistant and associated with poor outcomes (Bach et al., 2009; Leitman et al., 2010, 2011). To advance our understandings of the relationship between the symptoms of schizophrenia and the perception of emotional voices, this study, through the neurophysiological approach, clarified whether emotional voice processing is impaired *per se*, and further, associated with sensory dysfunction or attention abnormalities.

The extent to which basic auditory processing contributes to impaired voice perception in schizophrenia is unclear. Some studies reported that deficits of emotional prosodic identification in individuals with schizophrenia reflect, at least in part, a relative inability to process the acoustic characteristics of prosodic stimuli (Leitman et al., 2005, 2010, 2011). They have argued that schizophrenia is associated with structural and functional disturbances at the primary auditory cortex (Leitman et al., 2007). However, other studies found that individuals with schizophrenia had more difficulties at emotional prosody comprehension than controls, but equivalently proficient at stress prosody comprehension (Murphy and Cutting, 1990). Their performance was worse at identifying high-clarity emotional prosodic stimuli, but not at identifying low-clarity stimuli (Bach et al., 2009). Individuals with schizophrenia relative to healthy controls showed comparable performance for discriminating among terminal pitch changes, but more difficulties for internal pitch discrimination (Matsumoto et al., 2006).

Mismatch negativity (MMN) and P3a are event-related potentials (ERPs) that can be elicited by a passive oddball paradigm. MMN and P3a have been used as neurophysiological biomarkers in schizophrenia research (Javitt et al., 2008; Javitt and Sweet, 2015). MMN reflects a preattentive stage of auditory information processing. For MMN generation, oddball stimuli may differ from standards based on a number of physical dimensions, including sensory modality, frequency, duration, or intensity (Näätänen et al., 2007). Primary generators for MMN are located in the primary auditory cortex (Alho, 1995; Maess et al., 2007). Through a meta-analysis, deficits in MMN generation were suggested to be a robust feature in chronic schizophrenia, indicating abnormalities in automatic context-dependent auditory information processing in these patients (Umbricht and Krljes, 2005). MMN reduction was associated with global impairments in everyday functioning in schizophrenia patients (Light and Braff, 2005). MMN appeared to be reduced, even at illness onset (Salisbury et al., 2002, 2007; Umbricht et al., 2006; Jahshan et al., 2012). In addition, P3a is an ERP-index of an involuntary attention switch (Escera et al., 2000). Auditory P3a is the earliest ERP abnormality to be studied in schizophrenia (Roth and Cannon, 1972). P3a was reduced in patients with chronic schizophrenia (Mathalon et al., 2000; Jeon and Polich, 2003). P3a might serve as a risk or trait marker of the genetic risk of schizophrenia (Winterer, 2000; Hall et al., 2006).

Until recently, emotional MMN and P3a were not utilized to assess the automaticity and involuntary attention of emotional salience processing of voices, respectively (Schirmer et al., 2005). The unexpected presence of emotionally spoken syllables embedded in a passive oddball paradigm can trigger emotional MMN and P3a. Particularly, emotional mismatch response, an infant analog of the adult emotional MMN, was identified in newborns, reflecting the emergence of emotional arousal during the first days of life (Cheng et al., 2012). Females exhibited stronger emotional MMN and P3a than did males, inferring the sex hormone-mediated processing of emotional voices (Hung and Cheng, 2014). Testosterone administrations could alter emotional MMN and P3a, lending support to the involvement of amygdala in the generator sources (Chen et al., 2015). These findings support the notion that emotional MMN and P3a can probe emotional voice processing. In the same vain, emotional MMN and P3a were reduced in individuals with autism spectrum conditions and lower angry MMN amplitudes were associated with higher levels of autistic traits (Fan and Cheng, 2014). However, to the best of knowledge, emotional MMN and P3a have been examined in individuals with schizophrenia.

To understand the extent to which basic auditory processing contributes to impaired emotional salience processing of voices, we presented the emotionally spoken meaningless syllables *dada*, and acoustically matched non-vocal sounds in a passive oddball paradigm to individuals with chronic schizophrenia and matched controls. It is worth to mention that the disrupted activity in amygdala might lead to abnormal assignment of salience to ambiguous, potentially threatening stimuli, such as angry voices, in patients with schizophrenia, particularly in those with positive symptoms (Holt and Philops, 2009). One neuroimaging study demonstrated that the amygdala was activated by using a passive oddball paradigm on angry syllables (Schirmer et al., 2008). It is thus hypothesized that, if general deficits in auditory processing existed, then patients with schizophrenia would exhibit altered MMN and P3a responses to angry and happy syllables and corresponding non-vocal sounds. If the deficit were selective for threatening voices, then individuals with schizophrenia would elicit distinct MMN and P3a to angry syllables from controls. In addition, to further explore the relationship between neurophysiological responses and symptom severity, we conducted correlation analyses to test the extent to which emotional MMN and P3a covaried with the Positive and Negative Syndrome Scale (PANSS).

## Materials and Methods

### Subjects

Thirty schizophrenia patients and thirty controls were enrolled. Individuals with schizophrenia were recruited from local hospital. Using the Structured Clinical Interview from the Diagnostic and Statistical Manual of Mental Disorders Fourth Revised Patient Edition, psychiatrists reconfirmed that the illness was in a non-acute and stable phase. All subjects were ethnic Chinese. The age- and handedness-matched controls were recruited from local community and screened for major psychiatric illnesses by using the Structured Clinical Interview for DSM-IV Axis I Disorders (SCID-I). **Table 1** lists demographics and clinical variables. Subjects with comorbid psychiatry or neurological disorders (e.g., dementia or seizures), a history of head injury, alcohol or substance abuse or dependence were excluded. All of the participants exhibited normal peripheral hearing bilaterally (pure tone average thresholds<15 dB HL) at the time of testing. For the handedness, medications, and does for each patient, please refer to **Table 2**. All subjects provided written informed consent and assent for the study, which was approved by local ethics committee (Yang–Ming University Hospital) and conducted in accordance with the Declaration of Helsinki.

**Table 1:** Demographic and clinical variables related to study participants.

	Schizophrenia *N* = 30 9 males		Controls *N* = 30 11 males		
	Mean	*SD*	Mean	*SD*	*p*-value
Age (yrs)	39.8	4.8	36.5	4.9	0.14
Education (yrs)	11.8	2.6	14.0	3.4	0.006
Duration of illness (yrs)	14.6	9.1			
PANSS (range)					
Positive scale (8 ~ 21)	14.1	3.7			
Negative scale (7 ~ 24)	14.2	3.8			
General Psychopathology (20 ~ 43)	31.4	6.1			

*Abbreviations: yrs, years; PANSS, the Positive and Negative Syndrome Scale (Kay et al., 1987; Phillips et al., 1991).*

**Table 2:** Handedness and medications for each individual with schizophrenia.

Subject	Right Handedness	Drug	Dose	Dose No.
F01	90%	Risdone	2 mg	1
		Clonopam	0.5 mg	0.25
		Minlife (Loratadine)	10 mg	1
F02	90%	Xanax (Alprazolam)	0.5 mg	1+0.5
		Clopine	100 mg	0.5+1
F03	80%	Bipiden (Biperiden)	2 mg	0.5
		Polupi	50 mg	1
		Risdone (Risperidone)	2 mg	1
		Januvia F.C	100 mg	1
		Cipram (Citalopram)	20 mg	0.5
		Eltroxin (Thyroxine Sodium)	100 mg	1.5
		Imovane (Zopiclone)	7.5 mg	0.5
F04	85%	Risdone	2 mg	1
		Zolman.F.C	10 mg	2
		Bipiden	2 mg	1
		Clonopam	0.5 mg	1
		venforspine	75 mg	1
		Rivotril	2 mg	1
		Etomine	40 mg	2
F05	90%	Juxac (Fluoxetine)	20 mg	1
		Olanzapine	5 mg	1
		Delopic	20 mg	1
F06	90%	Juxac (Fluoxetine)	20 mg	
		Mesyrel (Trazodone HCl)	50 mg	1
		Clonopam (Clonazepam)	0.5 mg	0.5
		Abilify (Aripiprazole)	10 mg	1
		Zolman	10 mg	1
F07	85%			
F08	90%			
F09	100%	Fluanxol	3 mg	1
F10	100%	Clopine	100 mg	2
F11	100%	Clopine	100 mg	1
		Trynol S.C	25 mg	1
F12	100%	Ativan (Lorazepam)	0.5 mg	1
		Dupin (Diazepam)	5 mg	0.5
		Inderal (Propranolol)	10 mg	1
		Zyprexa Zydis	5 mg	1
F13	90%	Xanax	0.5 mg	1
		Fluanxol	3 mg	2
		Etumine	40 mg	1
		Clonopam	0.5 mg	1
		Rivotril	2 mg	0.5
		Bipiden	2 mg	1
F14	85%	Clopine	100 mg	1.5
		Semi-Nax	10 mg	1
		Clonopam	0.5 mg	0.5
		Juxac	20 mg	1
F15	65%	Semi-Nax	10 mg	1
		Xanax	0.5 mg	0.5
		Zyprexa Zydis	5 mg	1.5
F16	75%	Xanax	0.5 mg	1
		Modipan	2 mg	1
		Dogmatyl	200 mg	3
		Efexor	37.5 mg	1
		Clonopam	0.5 mg	0.5
		Morefine	100 mg	1
		Parlodel	2.5 mg	0.5
F17	100%	Abilify	10 mg	0.5
		Silence	1 mg	1
F18	80%	Bipiden	2	1
		Flurazin	5	1
		Juxac	20	1
		Lexotan	3	0.25
F19	100%	Abilify	10	1.5
		Rivotril	2	0.5
		Bipiden	2	0.5
F20	85%	Semi-Nax	10 mg	0.5
		Clonopam	0.5 mg	0.5
		Risdone	2	1
F21	90%			
M01	85%	Fluanxol	3 mg	1
		Silence	1 mg	1
		Bipiden	2 mg	1
		Etumine	40 mg	1
		Clonopam	0.5 mg	1
		Fluanxol	20 mg/1 ml	1
		Juxac	20 mg	1
M02	100%	Clopine (Clozapine)	100 mg	2
		Crestor	10 mg	1
M03	100%	Clopine	100 mg	1
		Clonopam	0.5 mg	0.5
		Juxac	20 mg	1
		Semi-nax	100 mg/tab	1
		Xanax	0.5 mg	0.5
		Vitacome		1
		Lipanthyl	200 mg	1
M04	85%			
M05	80%	Dogmatyl (Sulpiride)	200 mg	1
		Genbou F.C (Fluvoxamine)	50 mg	1
		Zyprexa Zydis	5 mg	1.5
M06	90%			
M07	90%	Dogmatyl	200 mg	2
		Bipiden	2 mg	1.5
M08	90%	Bipiden	2 mg	1
		Zolman F.C	10 mg	1
		Eurodin	2 mg	1
		Clonopam	0.5 mg	1
		Dogmatyl	200 mg	3
M09	85%	Bipiden	2 mg	1
		Dogmatyl	2 mg	1

### Auditory Stimuli

The stimuli have two categories: emotional syllables and acoustically matched non-vocal sounds. For emotional syllables, a young female speaker from a performing arts school produced the meaningless syllable *dada* with three sets of emotional (angry, happy, neutral) prosodies. Within each set of emotional syllables, the speaker produced the syllables for more than ten times. Emotional syllables were edited to become equally long (550 ms) and loud (min: 57 dB; max: 62 dB; mean 59 dB) using Cool Edit Pro 2.0 and Sound Forge 9.0. Each syllable set was rated for emotionality on a 5-point Likert-scale (see Cheng et al., 2012; Fan et al., 2013; Fan and Cheng, 2014 for validation). Two emotional syllables that were consistently identified as ‘extremely angry’ and ‘extremely happy’ and one neutral syllables rated as the most emotionless were selected as the stimuli. The ratings on the Likert-scale (mean ± SD) were 4.26 ± 0.85, 4.04 ± 0.91, and 2.47 ± 0.87 for angry, happy, and neutral syllables, respectively.

**FIGURE 1:**
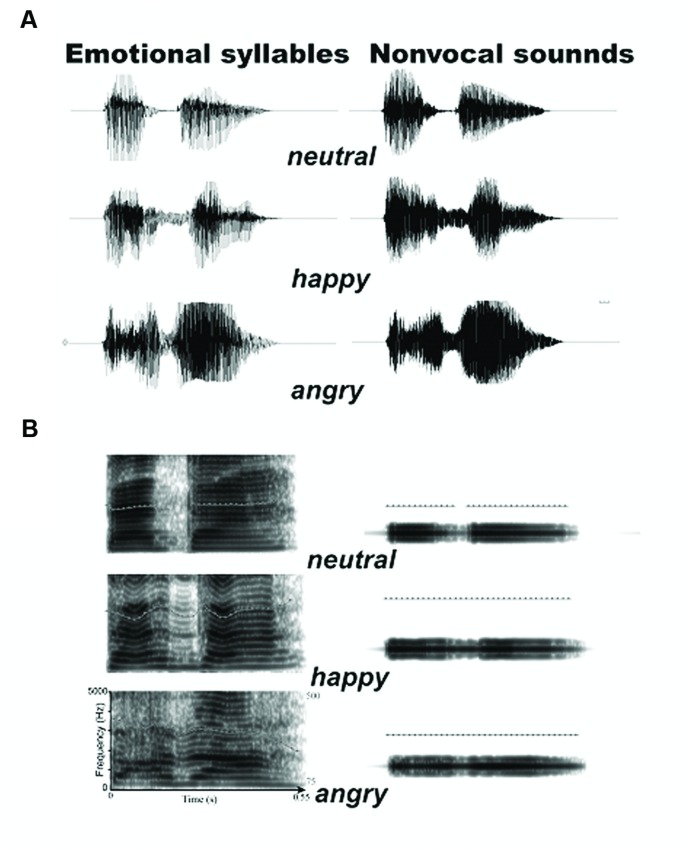
**Acoustic properties of stimulus materials. (A)** Oscillogram of auditory stimulus. **(B)** Spectrogram of auditory stimulus. Non-vocal sounds retain the spectral centroid (fn) as well as the temporal envelope of emotional syllables.

To create a set of control stimuli that retain acoustical correspondence with emotional syllables, we synthesized non-vocal sounds by using Praat (Boersma, 2001) and MATLAB (The MathWorks, Inc., USA). The central gravity of frequency (fn) of each original syllable was defined as [|∫ X(f)|^2^× f *d*f)/(|∫ X(f)|^2^ *d*f], where X(f) was the Fourier spectrum of emotional syllables. The fn of angry, happy, and neutral syllables was 1249 Hz, 1159 Hz, and 1156 Hz, respectively. We then produced non-vocal sounds by multiplying the sine waveform with two Hamming windows that were temporarily centered at each of the syllable [non-vocal sounds = fn(t) × Hamming window(t)]. This way has been used to synthesize non-vocal sounds for controlling the temporal envelope and core spectral element of emotional syllables (Fan et al., 2013; Chen et al., 2014; Hung and Cheng, 2014). The time-course and frequency spectrum of emotional syllables and corresponding non-vocal sounds are illustrated in **Figure 1**. In addition, non-vocal sounds had comparable emotionality ratings on the Likert-scale (2.47 ± 0.87) with neutral syllables (*P* > 0.1) as well as below-chance hits on the emotional categorization task (Chen et al., 2014), indicating emotional neutrality of acoustic controls.

### EEG Apparatus, Procedures, Recording, and Data Analysis

Before EEG recordings, psychiatrists administered the PANSS (Kay et al., 1987; Phillips et al., 1991) to evaluate the symptom severity of schizophrenia. The EEG recording was conducted in a sound-attenuated and electrically shielded room. During EEG recording, participants were required to watch a silent movie with subtitles while task-irrelevant stimuli in oddball sequences were presented. Particularly, instead of presenting physically identical stimuli as both of standards and deviants (Schirmer et al., 2007), we applied the same theorem as previous work for controlling the mismatch paradigm (Čeponienë et al., 2003; Chen et al., 2014). The passive oddball paradigm for emotional syllables employed happy and angry syllables as deviants (D1, D1) and neutral syllables as standards (S). Their correspondingly non-vocal sounds were applied in the same oddball paradigm, but were presented as separate blocks so that relative acoustic features among S, D1, and D2 were controlled across blocks. Each stimulus category (emotional syllables vs. non-vocal sounds) comprised two blocks, the order of which was counter-balanced and randomized across participants. Each block consisted of 600 trials, of which 80% were neutral syllables or neutral-derived sounds, 10% were angry syllables or angry-derived sounds, and 10% were happy syllables or happy-derived sounds. The sequences of blocks and stimuli were quasirandomized to avoid successive blocks and successive deviants from identical stimulus categories. A minimum of two standards was always presented between any two deviants. The stimulus-onset-asynchrony was 1200 ms, including a stimulus length of 550 ms and an inter-stimulus interval of 650 ms.

The MMN and P3a amplitudes were analyzed as an average within a 50-ms window surrounding the peak at selected electrode sites. Based on prior literature (Näätänen et al., 2007, 2011), the MMN peak was defined as the largest negativity after subtracting the standard ERP from the deviant ERP during a period of 150 to 350 ms after stimulus onset. Only the standards before the deviants were included in the analysis. The P3a peak was defined as the largest positivity within the period of 250 to 450 ms. Three-way mixed ANOVAs were separately performed on MMN and P3a for each category (emotional syllables or non-vocal sounds) with deviant type (happy or angry) and electrode site (F3, Fz, F4, C3, Cz, or C4) as the within-subject factors and group (schizophrenia or control) as the between-subject factor. The dependent variables were the mean amplitudes and peak latencies of the MMN and P3a components at the selected electrode sites. Degrees of freedom were corrected using the Greenhouse–Geisser method while sphericity had been violated. A Bonferroni-corrected *t-*test was only conducted when preceded by significant main effects. Spearman’s correlation analysis was conducted between emotional MMN or P3a and the PANSS subscales.

## Results

### Neurophysiological Measures between Groups

**FIGURE 2:**
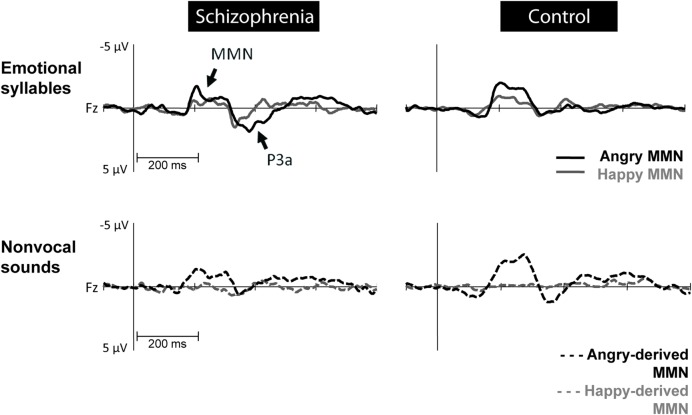
**Mismatch negativity (MMN) amplitudes to emotional syllables and non-vocal sounds at electrode site Fz between individuals with schizophrenia and their matched controls.** Non-vocal deviants retaining acoustical features of emotional syllables were derived from angry (angry-derived) and happy (happy-derived) syllables. MMN to angry and angry-derived deviants (black solid and dotted lines) were significantly weaker in individuals with schizophrenia than in the controls (*p* = 0.02; *p* = 0.007), whereas no differences between these two groups emerged in the MMN to happy and happy-derived deviants (gray solid and dotted lines).

Preattentive discrimination of emotional voices was studied using MMN, determined by subtracting neutral ERP from angry and happy ERPs (**Table 3**). The ANOVA on MMN amplitudes to emotional syllables reflected main effects exerted by the deviant type [*F*(1,58) = 70.60, *p* < 0.001, $\eta_{p}^{2}$ = 0.55], electrode [*F*(1,58) = 4.97, *p* < 0.001, $\eta_{p}^{2}$ = 0.08], and group [*F*(1,58) = 4.45, *p* = 0.039, $\eta_{p}^{2}$ = 0.07] (**Figure 2**). As compared to controls (mean ± SE: 1.9 ± 0.16 μv), individuals with schizophrenia displayed weaker emotional MMN (1.44 ± 0.16) irrespective of happy or angry deviants. Angry MMN (2.15 ± 0.14) was significantly stronger than happy MMN (1.19 ± 0.11) irrespective of schizophrenia or control. MMN had the strongest amplitudes at electrode *F*z (1.83 ± 0.13) as compared to F3 (1.68 ± 0.11), F4 (1.72 ± 0.12), C3 (1.57 ± 0.1), Cz (1.66 ± 0.13), or C4 (1.56 ± 0.11). In addition, none of their interactions reached significance, including electrode × group [*F*(5,290) = 1.84, *p* = 0.132, $\eta_{p}^{2}$ = 0.03], deviant type × group [*F*(1,58) = 1.11, *p* = 0.296, $\eta_{p}^{2}$ = 0.02], electrode × deviant type [*F*(5,290) = 0.19, *p* = 0.914, $\eta_{p}^{2}$ < 0.01] or electrode × deviant type × group [*F*(5,290) = 1.72, *p* = 0.160, $\eta_{p}^{2}$ = 0.03].

**Table 3:** Mean amplitude and latencies of MMN to emotional syllables and non-vocal sounds within a time window of 150 to 350 ms at frontal electrodes in individuals with schizophrenia and controls (Mean ± SD).

			Schizophrenia		Controls	
			Amplitudes (μV)	Latency (ms)	Amplitudes (μV)	Latency (ms)
Emotional syllables	Angry	F3	-1.77 ± 1.20	245.27 ± 57.25	-2.55 ± 1.12	251.00 ± 51.81
		Fz	-1.94 ± 1.23	239.33 ± 54.31	-2.69 ± 1.19	247.93 ± 55.60
		F4	-1.85 ± 1.30	244.87 ± 52.14	-2.58 ± 1.02	256.27 ± 55.67
	Happy	F3	-1.04 ± 0.94	245.33 ± 49.34	-1.33 ± 0.68	259.33 ± 51.61
		Fz	-1.17 ± 1.18	248.93 ± 51.43	-1.51 ± 0.76	255.47 ± 51.24
		F4	-1.01 ± 1.10	244.21 ± 40.41	-1.45 ± 0.75	258.33 ± 47.54
Non-vocal sounds	Angry-derived	F3	-1.87 ± 1.15	245.13 ± 47.81	-2.65 ± 1.16	251.13 ± 36.40
		Fz	-2.06 ± 1.14	245.00 ± 47.28	-2.89 ± 1.18	249.00 ± 37.76
		F4	-2.00 ± 1.00	237.93 ± 47.46	-2.73 ± 1.20	248.07 ± 37.65
	Happy-derived	F3	-0.75 ± 0.76	233.67 ± 54.83	-0.76 ± 0.53	262.13 ± 56.30
		Fz	-0.64 ± 0.85	241.87 ± 50.72	-0.89 ± 0.57	261.60 ± 54.61
		F4	0.67 ± 0.91	251.47 ± 56.04	-0.79 ± 0.55	253.07 ± 55.45

Mismatch negativity amplitudes to non-vocal sounds showed main effects for the deviant type [*F*(1,58) = 132.58, *p* < 0.001, $\eta_{p}^{2}$ = 0.70], electrode [*F*(1,58) = 11.75, *p* < 0.001, $\eta_{p}^{2}$ = 0.17], and group [*F*(1,58) = 5.47, *p* = 0.023, $\eta_{p}^{2}$ = 0.09], as well as interactions for deviant type × electrode [*F*(1,58) = 8.02, *p* < 0.001, $\eta_{p}^{2}$ = 0.12] and deviant type × group [*F*(1,58) = 5.42, *p* = 0.023, $\eta_{p}^{2}$ = 0.09] (**Figure 2**). *Post hoc* analyses revealed that angry-derived MMN exerted an electrode effect (*p* < 0.001) with the strongest amplitude at the electrode site Fz (2.47 ± 0.15 μv), but happy-derived MMN did not exhibit this effect (*p* = 0.17). Individuals with schizophrenia relative to the controls showed weaker angry-derived MMN (*p* = 0.037) but comparable happy-derived MMN (0.75 ± 0.12 vs. 0.65 ± 0.12, *p* = 0.56).

Mismatch negativity peak latencies to emotional syllables and non-vocal sounds did not reveal any effect involving the group factor. It indicated that individuals with schizophrenia did not differ from controls in term of the speed of preattentive processing.

Involuntary attention switches to emotional voices were studied using P3a (**Table 4**). P3a amplitudes to emotional syllables reflected main effects for the deviant type [*F*(1,58) = 4.66, *p* = 0.035, $\eta_{p}^{2}$ = 0.07] and electrode [*F*(1,58) = 7.60, *p* < 0.001, $\eta_{p}^{2}$ = 0.12] as well as a statistically non-significant trend toward the group effect [*F*(1,58) = 3.01, *p* = 0.088, $\eta_{p}^{2}$ = 0.05]. As compared to controls (1.13 ± 0.18 μv), individuals with schizophrenia displayed stronger emotional P3a to emotional syllables (1.58 ± 0.18) irrespective of happy or angry deviants. Angry P3a (1.52 ± 0.16 μv) was stronger than happy P3a (1.19 ± 0.14) irrespective of group or electrode. P3a had the strongest amplitude at Fz (1.54 ± 0.15) as compared to F3 (1.45 ± 0.14), F4 (1.37 ± 0.14), C3 (1.21 ± 0.12), Cz (1.44 ± 0.16), or C4 (1.14 ± 0.13).

**Table 4:** Mean amplitude and latencies of P3a to emotional syllables and non-vocal sounds within a time window of 250 to 450 ms at frontal electrodes in individuals with schizophrenia and controls (Mean ± SD).

			Schizophrenia		Controls	
			Amplitudes (μV)	Latency (ms)	Amplitudes (μV)	Latency (ms)
Emotional syllables	Angry	F3	2.03 ± 1.28	376.87 ± 46.66	1.22 ± 1.44	362.67 ± 51.43
		Fz	2.09 ± 1.54	378.53 ± 47.61	1.41 ± 1.45	354.87 ± 48.45
		F4	1.90 ± 1.51	384.73 ± 46.95	1.20 ± 1.18	350.13 ± 49.48
	Happy	F3	1.36 ± 1.78	341.40 ± 45.50	1.19 ± 1.04	346.27 ± 47.25
		Fz	1.43 ± 1.41	340.53 ± 47.29	1.24 ± 1.10	358.27 ± 46.95
		F4	1.30 ± 1.37	346.67 ± 47.87	1.08 ± 0.90	357.93 ± 50.15
Non-vocal sounds	Angry-derived	F3	0.82 ± 1.13	363.73 ± 45.60	1.28 ± 1.24	366.67 ± 29.31
		Fz	0.96 ± 1.19	356.20 ± 50.27	1.41 ± 1.38	366.93 ± 27.82
		F4	0.73 ± 1.03	365.73 ± 51.01	1.32 ± 1.20	361.60 ± 25.85
	Happy-derived	F3	0.93 ± 0.95	338.93 ± 52.45	0.69 ± 0.69	349.33 ± 56.44
		Fz	1.16 ± 1.33	334.20 ± 46.04	0.77 ± 0.68	340.67 ± 59.35
		F4	1.08 ± 1.22	338.47 ± 45.90	0.79 ± 0.63	348.07 ± 53.53

P3a amplitudes to non-vocal sounds showed an electrode effect [*F*(1,58) = 5.09, *p* < 0.001, $\eta_{p}^{2}$ = 0.03] and an deviant type × group interaction [*F*(1,58) = 5.32, *p* = 0.025, $\eta_{p}^{2}$ = 0.08]. *Post hoc* analyses indicated that angry-derived P3a was stronger than happy-derived P3a for control [*F*(1,58) = 4.93, *p* = 0.034, $\eta_{p}^{2}$ = 0.15], whereas no difference emerged for schizophrenia [*F*(1,58) = 0.79, *p* = 0.38, $\eta_{p}^{2}$ = 0.03].

P3a peak latencies to emotional syllables reflected an effect for the deviant type [*F*(1,58) = 14.12, *p* < 0.001, $\eta_{p}^{2}$ = 0.20] and an deviant type × group interaction [*F*(1,58) = 5.47, *p* = 0.023, $\eta_{p}^{2}$ = 0.09]. *Post hoc* analyses indicated that schizophrenia had longer latencies for angry P3a than controls [*t*(58) = 2.27, *p* = 0.027, *d* = 0.59], whereas two groups displayed similar happy P3a [*t*(58) = 0.74, *p* = 0.46, *d* = 0.19]. In addition, P3a peak latencies to non-vocal sounds reflected an effect for the deviant type [*F*(1,58) = 11.51, *p* = 0.001, $\eta_{p}^{2}$ = 0.17], indicating earlier latencies for happy-derived P3a than angry-derived P3a irrespective of group or electrode.

### Correlation between Emotional MMN or P3a and Symptom Severity

**FIGURE 3:**
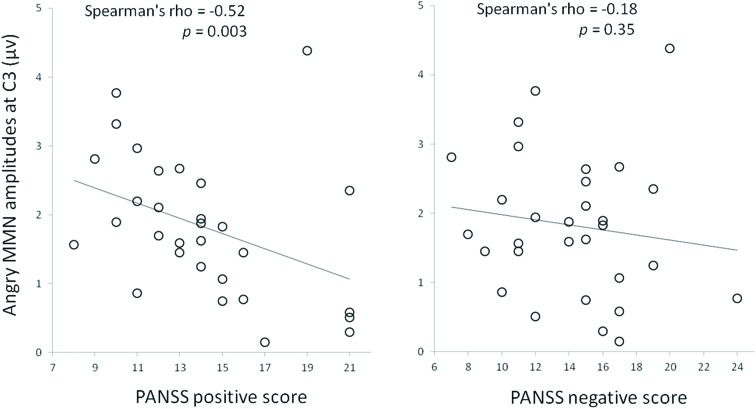
**Correlations between emotional MMN amplitudes and symptom severities in individuals with schizophrenia.** The severity of positive and negative symptoms was assessed with the PANSS.

There were significant correlations between angry MMN amplitudes and positive symptoms (**Table 5**). The Holm–Bonferroni step-down procedure was conducted to control the family wise error rate (FWER, *p* < 0.05) for multiple comparisons. Spearman’s correlation analyses on the PANSS subscales indicated that angry MMN amplitudes at C3 were negatively correlated with positive symptoms (ρ = -0.52, *p* = 0.003) (**Figure 3**). Such correlation was not observed either in the negative symptoms or general psychopathology scores. P3a was not correlated with the PANSS. In addition, neither MMN nor P3a exhibited any age-related correlation.

**Table 5:** The correlation analysis between PANSS (positive, negative, and general psychopathology) and angry MMN/P3a amplitudes, including the correlation coefficients (ρ) and *P* values corrected for multiple comparisons.

	F3	Fz	F4	C3	Cz	C4
**MMN**						
Positive scale						
ρ	-0.41	-0.36	-0.33	-0.52*	-0.42	-0.51
*P*	0.375	0.630	0.075	0.003	0.330	0.060
Negative scale						
ρ	-0.30	-0.05	0.04	-0.18	-0.13	-0.20
*P*	0.111	0.794	0.828	0.347	0.491	0.288
General psychopathology						
ρ	-0.17	-0.07	0.03	-0.27	-0.17	-0.26
*P*	0.357	0.718	0.890	0.145	0.378	0.169
**P3a**						
Positive scale						
ρ	0.01	-0.16	-0.22	-0.01	0.06	-0.12
*P*	0.953	0.452	0.274	0.945	0.767	0.545
Negative scale						
ρ	0.30	0.06	-0.11	-0.01	0.05	-0.17
*P*	0.130	0.773	0.596	0.975	0.824	0.401
General psychopathology						
ρ	0.09	-0.12	-0.21	0.07	0.05	-0.11
*P*	0.640	0.553	0.290	0.749	0.793	0.581

### Relationship between Sensitivity and Specificity for Angry MMN

**FIGURE 4:**
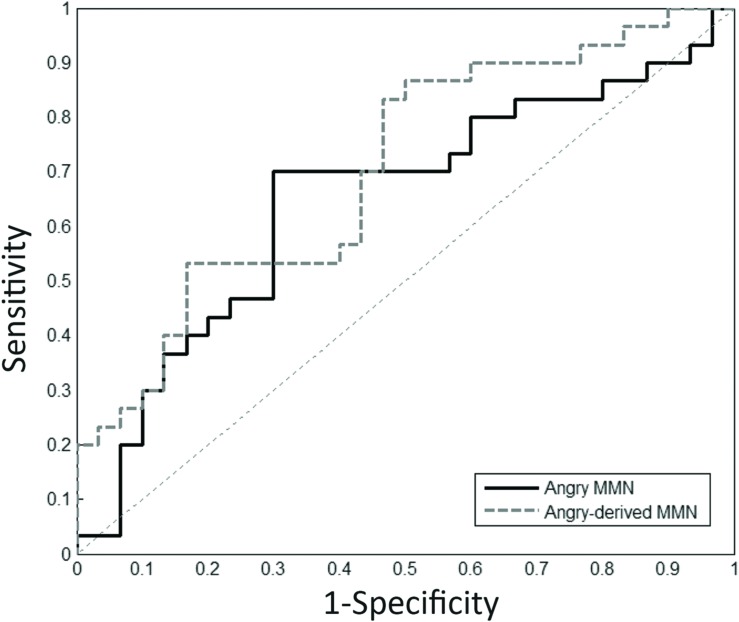
**Receiver operating characteristic (ROC) analysis.** Angry and angry-derived MMN amplitudes were used as a predictor to differentiate schizophrenia patients from the controls.

Receiver operating characteristic (ROC) analyses was conducted to measure the ability of emotional and non-vocal MMN amplitudes to differentiate between schizophrenia and control individuals (**Figure 4**). The area under the ROC curve (AUC) is indicative of the overall accuracy of the measure, representing the probability that a randomly selected true-positive individual scored higher on the measure than a randomly selected true-negative individual while 50% was chance level.

Receiver operating characteristic analysis for angry MMN resulted in AUC values of 0.65 (*p* = 0.049) over frontal electrodes. The most appropriate cut-off point for angry MMN with sensitivity of 70% and specificity of 70% was -1.89 μV. The AUC values for angry-derived MMN and angry P3a were 0.70 (*p* = 0.007) and 0.66 (*p* = 0.037). This indicated that angry and angry-derived MMN as well as angry P3a could help predict whether someone had received a clinical diagnosis of schizophrenia or not.

## Discussion

This study aims to clarify the extent to which basic auditory processing contributes to impaired emotional prosodic detection in schizophrenia. The results indicated that abnormal assignment of salience to threatening voices could help predict positive symptoms in schizophrenia. MMN, indexing preattentive detection of emotional salience of voices, was significantly reduced to angry syllables and angry-derived non-vocal sounds in schizophrenia. P3a, an index for selective attention control, showed greater amplitudes but longer latencies to angry syllables in schizophrenia. Weaker MMN amplitudes were associated with more positive symptoms of PANSS. ROC analyses suggested that angry MMN and P3a could predict whether someone had received a clinical diagnosis of schizophrenia or not.

Mismatch negativity amplitudes decreased for angry syllables and angry-derived non-vocal sounds in chronic schizophrenia. This finding might support the proposal that basic auditory processing abnormalities contribute to affective prosody dysfunction in schizophrenia (Leitman et al., 2005, 2007, 2010, 2011). Similarly, affective prosody recognition and MMN amplitudes elicited by infrequent high-pitched tones in the oddball paradigm were significantly associated (Jahshan et al., 2013). The emotional-derived non-vocal sounds in this study may partially reflect analog frequency (pitch) changes in pure tones. Studies on schizophrenia patients have reported decreased MMN in response to pitch deviants (e.g., Javitt et al., 1993, 1998; Catts et al., 1995). As indicated by reduced MMN in response to angry syllables and angry-derived non-vocal sounds, this study demonstrates that people with chronic schizophrenia process emotional voices in an atypical fashion at the preattentive level. Emotional voice processing abnormalities might be partially driven by impaired processing of low-level acoustic parameters.

Angry P3a, an index for selective attention control, had longer latencies in schizophrenia patients than in controls. Despite general consensus that P3a indexes attention switching to novel stimuli associated with psychopathology, the findings on increases or decreases of P3a amplitudes in psychotic patients are mixed (Javitt et al., 2008). Some reports have stated that patients at risk for schizophrenia exhibit weaker P3a (Mathalon et al., 2000; Jeon and Polich, 2003), whereas another found that stronger P3a was associated with an increased risk (Winterer, 2000). Atypical P3a might reflect pathological distractibility in chronic psychiatric patients (Escera et al., 2000). Emotional voices usually attract involuntary attention (Grandjean et al., 2008). Disturbed reciprocal fronto-limbic pathways might impair prefrontal dominance for controlling the hyperactive limbic system, resulting in failure to inhibit irrelevant information (Weinberger, 1987). Schizophrenia patients with auditory hallucination symptoms find it more difficult to control their selective attention, particularly in the presence of emotional distracters (Alba-Ferrara et al., 2013). In this study, the presence of P3a differentiation between angry and happy syllables along with the absence to differentiate angry-derived from happy-derived non-vocal P3a among schizophrenia patients could be ascribed to an imbalance of involuntary attention switching between emotional voices and acoustic attributes. Consistent with P3 being quantitative phenotypes (Winterer et al., 2003), ROC analyses indicated that angry P3a could help predict whether someone had received a clinical diagnosis of schizophrenia.

Positive symptoms coupled with angry MMN amplitudes within schizophrenia patients support the hypothesis that prosodic dysfunction may mediate the misattribution of auditory hallucination (David, 1994). Hearing voices is the most common type of hallucination associated with positive schizophrenia symptoms. Deficits of emotional prosodic perceptions were proposed as critical contributors to the formation of auditory hallucinations (Leitman et al., 2005; Rossell and Boundy, 2005). Patients experiencing auditory hallucinations were not as successful at recognizing prosodic cues as the non-hallucinating patients (Shea et al., 2007). Hallucinating patients exhibited reduced activations in the amygdala and insula when hearing crying sounds (Kang et al., 2009). Sensory gating deficits reflect the inability to filter out extraneous noise from meaningful sensory inputs (Freedman et al., 1987). They cause a cascade failure, rendering the malfunctioned limbic system unable to detect the emotional salience of incoming stimuli (Anticevic et al., 2012).

Some limitation of this study must be acknowledged. First, regarding sample homogeneity, the generalizability of the results may be limited because people with acute schizophrenia were not included. Second, the MMN recording here may not be state of the art. Unlike to the use of the stimuli as both of standards and deviants for controlling the mismatch paradigm (Schirmer et al., 2007), the MMN effect in this study may be potentially driven by physical stimulus characteristics. However, based on the same theorems as previous work (Čeponienë et al., 2003), we have conducted a series of studies to verify emotional and non-vocal MMN in the strict sense of disentangling emotional salience from physical properties (Cheng et al., 2012; Hung et al., 2013; Fan and Cheng, 2014; Chen et al., 2015). This may not be the optimal design, and future studies are warranted with a larger sample size, in which people with acute schizophrenia are recruited and stimuli with greater acoustic correspondence are included.

This study demonstrates that patients with chronic schizophrenia exhibited reduced MMN responses to both of angry syllables and non-vocal sounds, indicating general impairments of voice perception and acoustic discrimination. The atypical processing of emotional salience at the preattentive level might be partially driven by impaired processing of low-level acoustic parameters. The failure to tune their attention to contextually irrelevant stimuli of emotional voices could be ascribed to pathological distractibility. In particular, the MMN amplitudes to emotional voices predicted the severity of positive symptoms. These findings could provide evidence for bottom–up (i.e., perceptually based) cognitive remediation approaches, and indicate that emotional MMN and P3a can be potential neurophysiological endophenotypes of schizophrenia (Turetsky et al., 2007; Javitt and Sweet, 2015).

## Author Contributions

CC, P-YW, and YC took part in designing the study. CC and C-CL undertook the statistical analysis. C-CL and YC managed the literature search and wrote the first draft of the manuscript. All authors have contributed to and approved the manuscript.

## Conflict of Interest Statement

The authors declare that the research was conducted in the absence of any commercial or financial relationships that could be construed as a potential conflict of interest.
